# Optical Design of a Novel Wide-Field-of-View Space-Based Spectrometer for Climate Monitoring

**DOI:** 10.3390/s22155841

**Published:** 2022-08-04

**Authors:** Luca Schifano, Francis Berghmans, Steven Dewitte, Lien Smeesters

**Affiliations:** 1 Brussels Photonics (B-PHOT), Department of Applied Physics and Photonics, Vrije Universiteit Brussel, Pleinlaan 2, 1050 Brussels, Belgium; 2 Royal Meteorological Institute of Belgium, Avenue Circulaire 3, 1180 Brussels, Belgium; 3 Flanders Make, Pleinlaan 2, 1050 Brussels, Belgium; 4 Royal Observatory of Belgium, Avenue Circulaire 3, 1180 Brussels, Belgium

**Keywords:** climate change, greenhouse gases, space instrumentation, spectrometer, wide field of view, telescope, freeform optics

## Abstract

We report on a near-infrared imaging spectrometer for sensing the three most prominent greenhouse gases in the atmosphere (water vapor, carbon dioxide and methane). The optical design of the spectrometer involves freeform optics, which enables achieving exceptional performance and allows progressing well beyond the state-of-the-art in terms of compactness, field-of-view, and spatial resolution. The spectrometer is intended to be launched on a small satellite orbiting at 700 km and observing the Earth with a wide field-of-view of 120° and a spatial resolution of 2.6 km at nadir. The satellite will ultimately allow for improved climate change monitoring.

## 1. Introduction

Monitoring and mitigating climate change is of major societal importance worldwide. Climate change increases mortality [1]. In 2019, the World Health Organization (WHO) estimated that approximately 250,000 people will die annually between 2030 and 2050 due to climate change [2]. More recently, Thiery et al. published a study estimating that in comparison to people born in 1960, children born in 2020 are more likely to experience a two- to seven-fold increase in extreme events, under current climate policy pledges [3]. Science has indeed demonstrated that more frequent, intense, durable and larger heatwaves will arise over the next decades [3,4,5,6,7].

Various authorities, such as the European Commission and the World Meteorological Society (WMO), are superintending the monitoring and prevention of global warming. The political agenda has prioritized a set of measures, such as the achievement of the Sustainable Development Goals (SDG), of which Climate Action is the 13th [8]; the outcomes of the Paris Agreement (COP21) in December 2015 [9] and those of the follow-on COP26 [10] held in November 2021. In 2021, the Intergovernmental Panel on Climate Change (IPCC) issued the first part of its 6th Assessment Report, highlighting the importance of the Earth’s energy imbalance (EEI) in climate change [11]. This EEI is a measure of the rate of global climate change on interannual-to-decadal time scales. In addition, it is directly linked to other components of the climate system, including global ocean heat uptake, warming of the atmosphere, warming of the land and melting of ice.

The climate system is controlled by complex and delicate mechanisms. It can be influenced or forced by both natural (volcanic eruptions, solar radiation, etc.) and anthropogenic (aerosols, greenhouse gases, etc.) factors [11]. In essence, climate science is truly multidisciplinary. To monitor the different aspects of climate change, scientists have compiled a list of key elements that need to be observed on Earth, named the essential climate variables (ECVs). Presently, the Global Climate Observing System (GCOS) specifies 54 ECVs [12]. Studying these variables shows the effects of climate change on the different geospheres (atmosphere, biosphere, lithosphere, cryosphere, etc.). A few ECVs are of fundamental importance, as they are directly linked to climate change [13]. They can be summarized in two important groups: (1) Earth’s radiation budget and (2) greenhouse gases in the atmosphere. Greenhouse gases, such as carbon dioxide and water vapor capture, heat the atmosphere and directly impact the Earth’s radiation budget.

Our previous research efforts have already addressed innovative spaceborne instrumentation with the aim of improving climate change monitoring by means of a better assessment of the Earth’s radiation budget. These instruments include (1) a radiometer to measure the total incoming solar radiation and total outgoing terrestrial radiation [14], (2) a first camera to measure shortwave radiation [15], and (3) a second camera to measure longwave radiation [16]. These three instruments are part of our space mission project named the Earth Climate Observatory (ECO) (previously known as ASTERIX [17]). On the longer term, we foresee extending the ECO space mission to include a fourth instrument that will monitor the most prominent greenhouse gases in the atmosphere. Using these four instruments in synergy on a small satellite platform will allow acquiring the big picture of climate change by linking our measurements of the radiation at the top-of-atmosphere with the evolution of trace gases in the atmosphere.

This paper focuses on this fourth space-based instrument, which will consist of two parts: (1) a freeform telescope and (2) a spectrometer operating in the spectral range 1100–1700 nm, to sense the three most prominent greenhouse gases: water vapor, carbon dioxide and methane. Sensing these greenhouse gases is of fundamental importance in the context of climate change monitoring, as they have a direct influence on the Earth’s energy imbalance, which causes global warming [11,14]. Observation from space offers benefits that cannot be achieved from the ground, such as a global coverage and a daily revisit time, enabling the monitoring of the global distributions with high spatial and temporal resolutions, reducing uncertainties in estimations of these greenhouse gases and providing for a better understanding of their impacts on climate change [18,19,20].

We pursue monitoring gas absorption within the 1100–1700 nm wavelength range. The incoming solar radiation is absorbed and reflected during its interaction with the atmosphere, clouds and the Earth’s surface. Consequently, the reflection spectra that will be collected and measured by our spectrometer shall be influenced by the presence of the greenhouse gases. Each of the greenhouse gases has a different chemical structure, and thus a different molecular energy level diagram and different absorption properties, enabling their identification and concentration determination.

The use of freeform optics—i.e., optical elements with surfaces that hold neither translational nor rotational symmetry—in space-based spectrometer designs, enables excellent image quality, while decreasing the mass and volume of the optical system, as it minimizes the number of optical elements for an extended field of view (FOV) [21]. This makes freeform optics well suited for space applications, particularly when targeting compact designs that would fit inside a CubeSat (a few dm^3^), or more generally, a SmallSat platform.

Our manuscript is structured as follows. First, we define the scientific requirements and technical constraints of this novel fourth instrument, and we detail how we intend to improve on the state-of-the-art in space-based spectrometers (Section 2). The Results section of this paper (Section 3) describes the optical design and performance analysis of the full instrument. Section 4 puts our results in a broader perspective and discusses future short-term and long-term challenges. Section 5 closes the paper with a summary and conclusions.

## 2. Methods

### 2.1. State-of-the-Art Space-Based Spectrometers

The monitoring of greenhouse gases by Earth-observing space missions is not new at all, and constant scientific and technological effort has been made during the past decades to improve this monitoring, and our understanding of climate change. Monitoring is currently conducted using space-based spectrometers in the Low-Earth-Orbit. The first spectrometers use a whiskbroom configuration, scanning the Earth perpendicularly to their line of motion. These spectrometers notably include the Global Ozone Monitoring Experiment (GOME) [22], its follow-on GOME-2 [23], the Greenhouse Gases Observing Satellite (GOSAT) [24,25], and the SCanning Imaging Absorption spectroMeter for Atmospheric CartograpHY (SCIAMACHY) [26].

A change in paradigm consisted of the Ozone Monitoring Instrument (OMI) instrument, the first wide-field-of-view (WFOV) spectrometer using the pushbroom (staring) configuration [27]. OMI is an instrument composed of two main parts: (1) a telescope part, dedicated to the collection of photons, and (2) a spectrometer part, dedicated to the sensing of trace gases. The telescope is in fact a novel concept, patented in 1998. It enables imaging a very wide field of view on a rectangular slit [28] and features a swath of 2600 km and a spatial resolution of 13 km × 24 km, which was unprecedented at that time. As its name suggests, OMI is dedicated to ozone monitoring. For this purpose, the spectrometer operates in the spectral range 270–500 nm.

To extend the spectral range to other greenhouse gases, the concept was adapted to deliver its successor; the TROPOspheric Monitoring Instrument (TROPOMI), on board the Sentinel-5P space mission, a precursor mission for the future Sentinel-5 [29]. The TROPOMI telescope relays the collected light to a spectrometer comprising four spectroscopy channels, each of which is electronically split into bands, and covers three spectral bands: 270 nm–495 nm (UV/VIS), 710 nm–775 nm (NIR), and 2305 nm–2385 nm (SWIR).

The TROPOMI telescope makes use of freeform optics, which is one of the latest technologies exploited in the field of optical design, and which allows achieving exceptional performance with a wide FOV [30]. Freeform optics is now a common feature to achieve high-end performance and compact spectrometers, even for smaller fields of view, as, e.g., in the Compact Hyperspectral Air Pollution Sensor (CHAPS) spectrometer that monitors local pollution in urban areas with a narrow FOV of 15° [31]. Owing to the use of freeform optics, TROPOMI achieves a swath of 2600 km like its predecessor, but with an improved spatial resolution of 7 km × 7 km, which is about 2 to 3 times better than OMI. The concept of this telescope will be re-used on several future satellites (e.g., Sentinel-4 and Sentinel-5), and can sometimes be found under the names of TSBOA [32], UVN [33] and UVNS [34].

Like OMI and TROPOMI, we target the use of a telescope using two freeform mirrors with an aperture stop located between them. However, we aim to extend the FOV to 120° and to improve the spatial resolution to 5 km or smaller. This will enable us to link our results with measurements of the Earth’s radiation budget and Earth’s energy imbalance, which will be provided by the ECO space mission. In this way, measurements of the greenhouse gases can be directly linked to the measurements of changes in radiative fluxes in our atmosphere. Furthermore, whilst TROPOMI is a multi-channel instrument covering several spectral ranges in the ultraviolet, visible and near infrared bands, it does not cover the spectral range between 1100 and 1700 nm. By filling this spectral gap with our instrument, we will provide supplementary information for a better understanding of the impact of greenhouse gases on climate change.

### 2.2. Optical System Strategy

We target the development of a novel space-based spectrometer for the monitoring of the most prominent greenhouse gases. Our instrument is composed of two parts: (1) a telescope part that collects light and sends it to the spectrometer slit, and (2) a spectrometer part that diffracts light and focuses light onto the detector. More specifically, we aim to design it with the widest FOV (120°, which is 12° more than the state-of-the-art in this field), a spatial resolution of 5 km or smaller (the state-of-the-art being 5 km), and to cover the spectral range between 1100 and 1700 nm, which is not covered yet by the current state-of-the-art. We envision a maximal system volume of 1 CubeSat Unit (1 U = 1 dm^3^), which is also more compact than the state-of-the-art.

A spatial resolution of 5 km or better at nadir is quite challenging, considering that the instrument will be at a nominal altitude of 700 km and needs to operate with an extremely wide FOV of 120°. Additionally, as we target the use of a small satellite platform (a CubeSat or a SmallSat/PROBA-type platform), the spectrometer needs to be compact and designed with a minimal number of elements, requiring advanced optics. Therefore, and in view of ensuring good image quality, we have exploited freeform optics for the optical design of our instrument.

We selected the pushbroom configuration, such that the FOV is linear to retrieve the spatial information in one dimension, and the spectral information is encoded in the other dimension, yielding a two-dimensional (2D) image on a detector. For that purpose, we will use a custom detector with a pixel pitch of 15 µm to image each field—each spot—with 2 × 2 pixels. The telescope collects the light from a WFOV of 120°, and makes use of two freeform mirrors to correct for aberrations, while focusing the light onto the spectrometer slit.

Our spectrometer targets the sensing of greenhouse gases in the near-infrared (1100 nm–1700 nm) wavelength range—in particular, the three most prominent greenhouse gases feature spectral lines in this spectral region [35]:

• H${}_{2}$O: 1130 nm and 1400 nm;
• CO${}_{2}$: 1240 nm, 1420 nm, 1570 nm and 1610 nm;
• CH${}_{4}$: 1670 nm.

**Figure 1:**
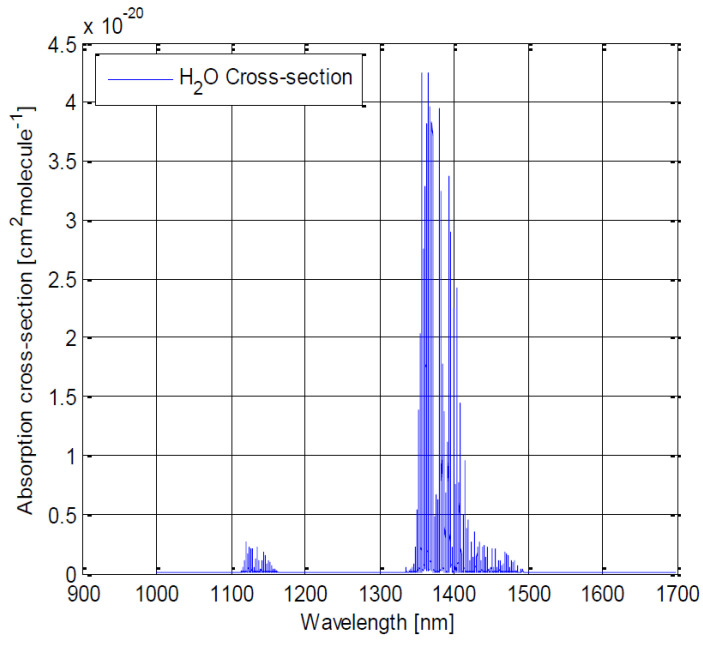
Absorption cross-section for H${}_{2}$O between 900 and 1700 nm. Key spectral lines are at 1130 and 1400 nm [36].

**Figure 2:**
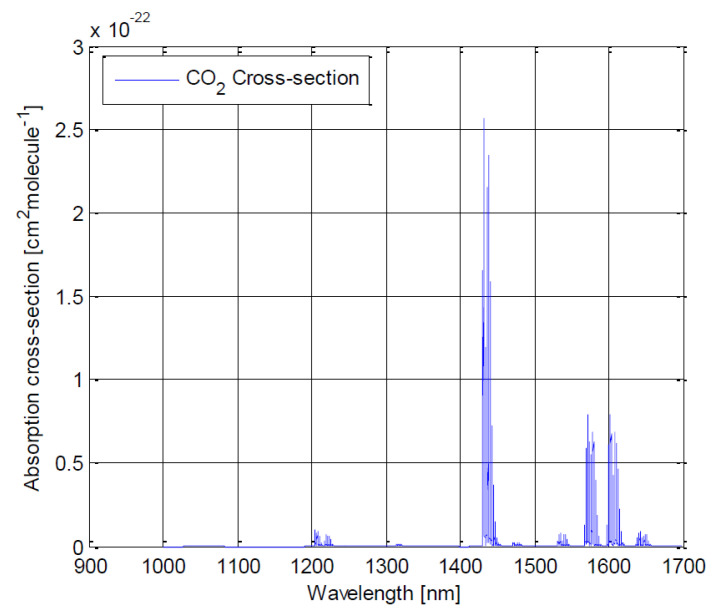
Absorption cross-section for CO${}_{2}$ between 900 and 1700 nm. Key spectral lines are at 1240, 1420, 1570 and 1610 nm [36].

**Figure 3:**
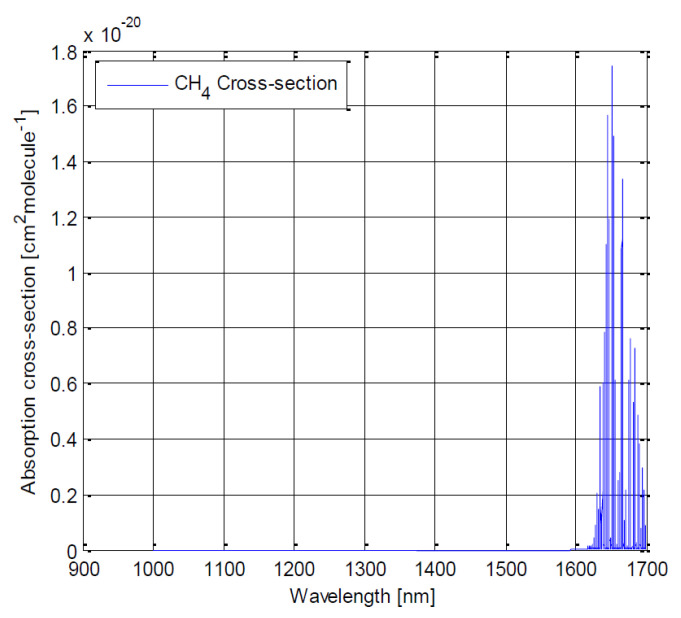
Absorption cross-section for CH${}_{4}$ between 900 and 1700 nm. Key spectral line is at 1670 nm [36].

As we aim to sense the envelope (Figure 1, Figure 2 and Figure 3), a spatial resolution of 20 nm (or shorter) would be preferable to sense and distinguish these three gases.

### 2.3. Optical Design Strategy

The optical design and analysis were performed using Zemax OpticStudio^®^ [37]. In the first step, we started by designing an on-axis telescope, including two mirrors and the slit after the secondary mirror, without any tilts, all the rays being in the same plane. The aperture stop is located between the two mirrors, and more specifically, at the focal point of the secondary mirror. Consequently, the rays leave the secondary mirror nearly parallel towards the spectrometer slit, allowing for telecentricity, which will in its turn ease the design of the spectrometer part. To maintain this telecentricity during optimization, the positions of the rays are constrained using the merit function. The merit function does thus not only contain the optical evaluation criteria that we pursue to optimize (e.g., minimizing the spot size and aberrations) but also includes beam steering conditions to minimize the differences between the real ray positions and the desired ray positions. The latter positions were selected with the view on telecentricity, to achieve parallel rays between the secondary mirror and the spectrometer slit.

We defined the F/# of our telescope such that an observation at 1.7 µm (upper band of our spectral range) corresponds to an Airy disk of 30 µm in diameter (two times the maximum pixel size that we are targeting for the detector to image each spot on 2 × 2 pixels). Using the Rayleigh criterion, our F/# equals 7.23, which is smaller—and thus more challenging—than the F/# of TROPOMI that is between 9 and 10.

We use the XY polynomials as mathematical descriptions of the freeform mirrors, as our telescope is off-axis with respect to the y-axis and features a symmetrical field of view with respect to the x-axis. We optimized the shape of our freeform mirrors, including their radii of curvature and XY polynomials until the 4th-order term (the first 24 XY polynomials). We first avoided using terms impacting the tilt of the mirrors. This way, the shape and the geometry of the mirrors were completely decorrelated, avoiding possible confusion. Once we obtained good image quality, we induced an incline of the mirrors with respect to the optical axis to avoid vignetting. It should be noted that this incline is forced using tilts of the mirrors rather than with decenters to reduce the keystone effect at the spectrometer slit. In addition, the keystone is reduced by constraining position of the rays on the detector in the merit function during the optimization process, which we did to maintain telecentricity. The exact shapes of the two mirrors and the distances between them and the spectrometer slit were found by a long iterative optimization process, involving trials with several configurations, including some telescope designs with three and four mirrors, and the use of other freeform terms. Finally, the telescope was optimized towards the most compact configuration.

After the spectrometer slit, a third freeform mirror collimates the light onto a custom reflective grating, featuring a moderate and feasible number of lines (120 lines per mm), allowing us to obtain the required spectral resolution of 20 nm. After the grating, two additional freeform imaging mirrors (radii of curvature = 132.98 mm and 166.32 mm) correct for aberrations for all wavelengths and all fields, and focus the collected light onto an area detector.

One dimension of the detector gives the spatial information and the second dimension the spectral information, implying that for every ground pixel coordinate and for each footprint, a reflection spectrum is obtained. This measured reflection spectrum is defined by the ratio of the Earth radiance spectra to the Sun irradiance spectra and the cosine of the solar Zenith angle [38]. Following that, hyperspectral processing techniques can be applied on the measurement data, including the application of chemometrics and machine learning to extract the information on the greenhouse gases’ concentrations. The optimization of these processing algorithms is a subject for future work, but will be based on the currently used spaceborne spectroscopy processing [39,40,41,42,43,44].

Further details about the spectrometer part, including grating, mirrors and detector, are given in Section 3.2.

## 3. Results

### 3.1. Optical Design and Performance Analysis of the Telescope Part

**Figure 4:**
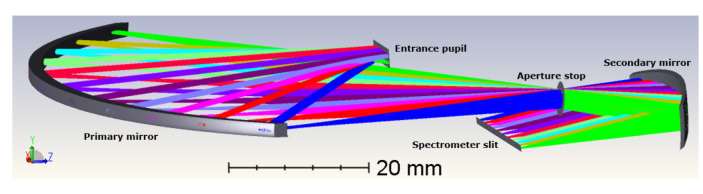
Optical design of our telescope. From top to bottom, the rays come from the entrance pupil (diameter = 2.52 mm), are reflected by the primary mirror (radius of curvature in X = 70.72 mm), go through the aperture stop (diameter = 2.94 mm), are reflected by the secondary mirror (radius of curvature in X = −35.82 mm) and finally reach the spectrometer slit (length = 26.4 mm). The different colors correspond to the different fields, from −60° to 60°, by step of 12°.

Our novel telescope (Figure 4) is compact (X, Y, Z = 96 mm × 15 mm × 95 mm), fitting within one CubeSat Unit (1 U = 100 mm × 100 mm × 100 mm). It is composed of two freeform mirrors arranged in an off-axis way. The aperture stop is located between the two mirrors, at the focal point of the secondary mirror. The primary mirror is the largest (96 mm in the X direction), enabling the collection of rays coming from a wide field of view of 120°. Telecentricity is achieved after the secondary mirror is turned towards the spectrometer slit.

**Figure 5:**
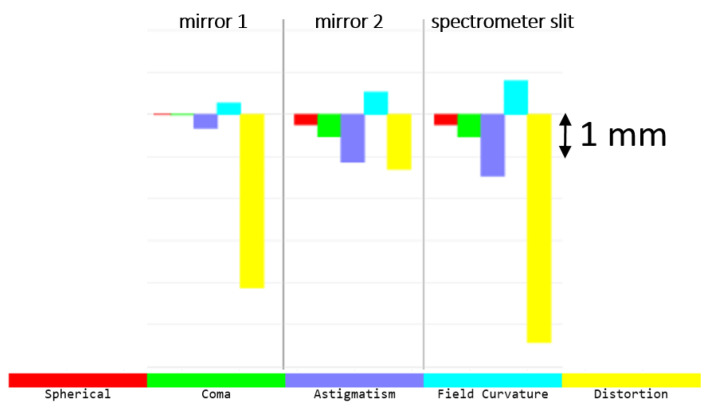
Contributions of the 5 Seidel aberrations. The largest aberration is the distortion, due to the large FOV. The aberrations are properly corrected, as the maximum scale is in the order of only 5 mm.

**Figure 6:**
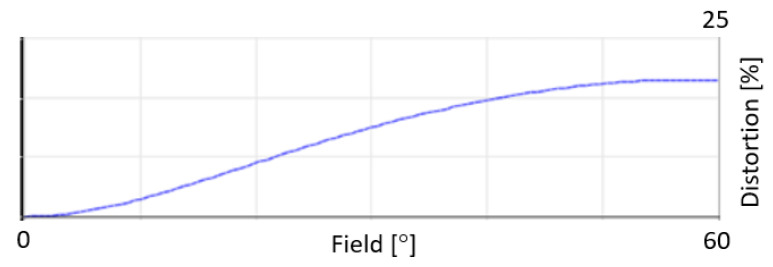
A close look at the (barrel) distortion. This aberration increases with the FOV to reach a maximum of 23% at ±60°.

Our optical design uses two freeform optics (XY polynomials until the 4th-order term) to correct for aberrations. Seidel aberrations include spherical aberration, coma, astigmatism, field curvature and distortion. As for the wide field of view, distortion is the largest aberration in the system (Figure 5). The primary mirror contributes the most to this aberration. However, the use of freeform optics effectively reduces all aberrations. At the spectrometer slit, the maximum aberration, being distortion, is in the order of 5 mm, and all other aberrations are less than 1.5 mm. A further look at the barrel distortion shows that this aberration increases with the FOV, reaching a maximum of 23% at ±60° (Figure 6). It is expected for WFOV telescopes to show such large distortion, but this aberration can be reduced after data acquisition by post-processing. Additionally, as a purely reflective design, the telescope does not suffer from chromatic aberrations.

**Figure 7:**
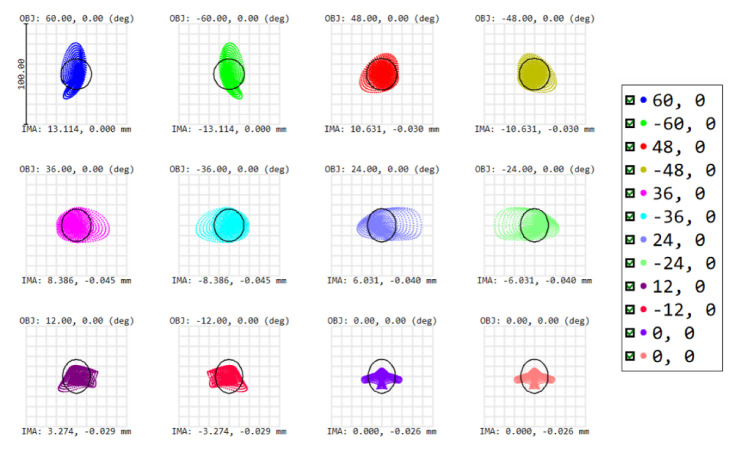
Spot diagram as the first figure of merit for our telescope optical performance. All RMS spot sizes are approximately the size of the Airy disk (indicated by the black circle) at a wavelength of 1700 nm, resulting in a nearly diffraction-limited design. Symmetry is properly achieved with respect to the 0°. OBJ (in degrees) defines the object field and IMA (in mm) defines the image height of the centroid on the detector. The different colors correspond to the different fields shown in Figure 4.

**Figure 8:**
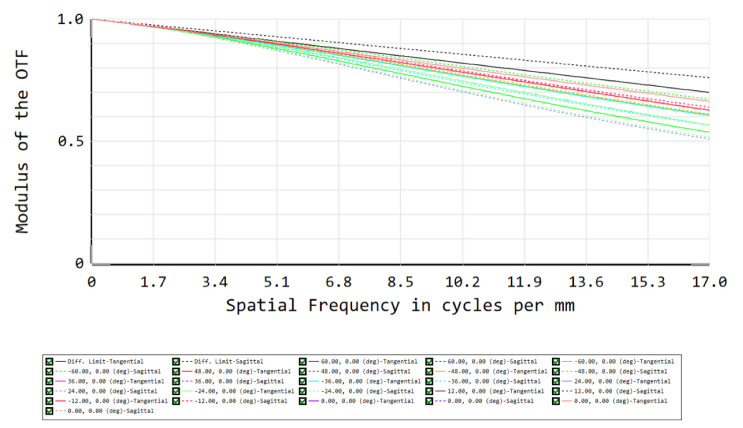
Modulation transfer function (MTF) as the second figure of merit for our telescope’s optical performance. The MTF exceeds 0.5 at 17 cycles/mm, indicating good image quality. The different colors correspond to the different fields shown in Figure 4.

To evaluate the system’s performance, our first figure of merit is the RMS spot size for each field, which should approximate the Airy disk radius, which equals 15 µm at the spectrometer slit. To meet this criterion at a wavelength of 1.7 µm, the F/# equals 7.23. After an iterative optimization process on the tilts, distances and XY polynomials, the spot diagram shows that all RMS spot sizes approximate the Airy disk—i.e., our optical design is close diffraction-limited (Figure 7). A further analysis of the optical performance can be achieved using a second figure of merit: the modulation transfer function (MTF), i.e., the modulus of the optical transfer function (OTF). To reach a good optical performance, we target a high MTF value at 17 cycles/mm, which is obtained for all fields (Figure 8).

In summary, the first part of our instrument is a telescope composed of two freeform mirrors that have been optimized using XY polynomials until the 4th-order term. The FOV equals 120°, which is 12° larger than the state-of-the-art (TROPOMI), and enables observation nearly from limb to limb. The optical performance is considered to be very good, using the spot diagram and MTF as figures of merit. On board of a satellite at a 700 km altitude, this telescope gives a spatial resolution of 2.6 km at nadir, which is 2.7 times better than the 7 km achieved by TROPOMI. The telescope is very compact and achieves telecentricity at the spectrometer slit, making it compatible with a compact spectrometer. In the next section, we focus on the optical design of the spectrometer part to achieve a full compact and performant design.

### 3.2. Optical Design and Performance of the Full Instrument Including the Spectrometer

In this section, we describe the design of the spectrometer part in order to obtain the full instrument. The spectrometer part follows the traditional architecture of dispersive imaging spectrometers for space applications: (1) the spectrometer slit, (2) a collimator (mirror or lens), (3) a dispersive element (a prism, a refractive or a reflective grating), (4) imaging mirror(s) or lens(es) and finally, (5) the detector.

Starting from the spectrometer slit, the incoming rays are actually the spots that were analyzed in the previous subsection. Consequently, rays leave the spectrometer slit nearly parallel owing to the telecentricity achieved with the telescope.

Rays are intercepted by a collimating mirror, which reflects the light onto the dispersive element. We favor the use of a reflective collimator instead of a refractive element for two reasons. First, it is easier to obtain a compact instrument. Second, we do not induce additional chromatic aberrations.

A freeform mirror is also used for the collimator. The light collimation is obtained in the YZ plane by choosing a radius of curvature in X of 100 mm, such that the focal point of the collimator is located at the spectrometer slit. Therefore, rays leaving the collimator are parallel in the direction perpendicular to the slit (Y-axis), reducing the divergence before arriving on the grating. This way, strong non-rotationally symmetric aberrations are avoided on the grating. Along the X-axis, we use freeform terms (X polynomials until the 4th-order term) to further correct for aberrations.

Next, rays arrive at the dispersive element. To keep the design compact, we selected a reflective diffraction grating, which is a periodic structure diffracting the light into different wavelength components. While the use of a transmission grating might be favorable for several reasons (high efficiency over a broad wavelength range, simple linear designs and less sensitive to alignment errors), we use a custom reflection grating (11.24 mm × 8.20 mm) because of the unavoidable tilt in the off-axis telescope. This reflective grating is composed of 120 lines/mm, resulting in the required spectral resolution of 20 nm with a moderate and feasible number of lines.

After the grating, two additional mirrors are used to image the light on the area of the detector. Freeform optics are used to correct for aberrations for all wavelengths. Again, these two mirrors are arranged in a way such that the instrument is as compact as possible. As for the telescope part, the mirrors in the spectrometer part are optimized according to the XY polynomials until the 4th-order term.

**Figure 9:**
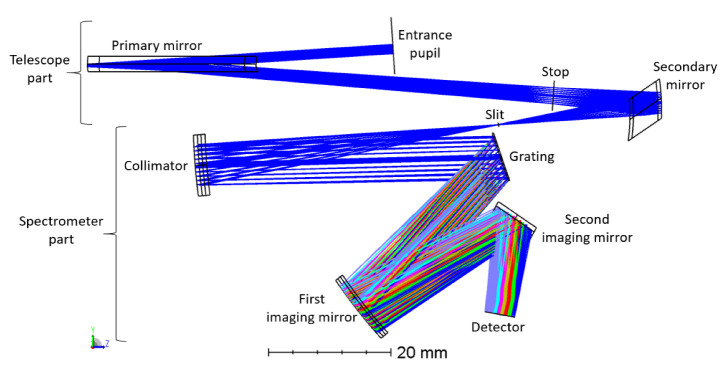
Overall optical design of the fully reflective instrument, featuring a freeform telescope with a field of view of 120° and a freeform spectrometer. The design is compact and fits within 1 dm^3^.

In total, the instrument is composed of five freeform mirrors and a reflective grating (Figure 9). The overall instrument is very compact to be compatible with a nanosatellite, as the full spectrometer optical design fits within 1 unit of CubeSat (1 dm^3^).

**Figure 10:**
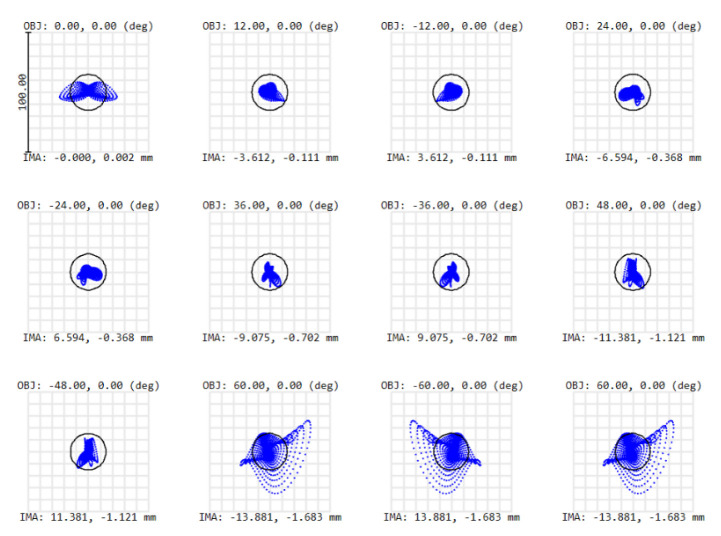
Spot diagram at 1700 nm over the full field of view. For each field, the root-mean-square size of the spot is smaller than the Airy disk (radius = 15 µm, indicated by the black circle), implying a diffraction-limited design. The results are shown for an equiangular sampling over the half field of view. Results are symmetrical for the other half part of the field of view. OBJ (in degrees) defines the object field, and IMA (in mm) defines the image height of the centroid on the detector.

**Figure 11:**
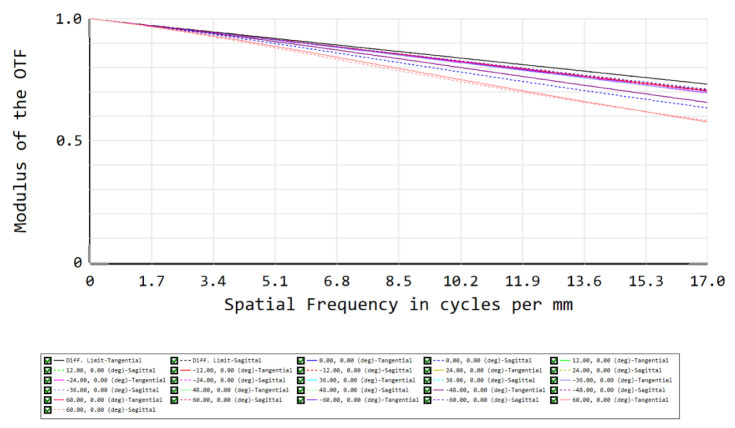
Modulation transfer function (MTF) at 1700 nm and at 17 cycles per mm over the full field of view, corresponding to the use of 2 × 2 pixels to image a spot of 30 µm in diameter. The MTF exceeds 0.5, which indicates an image quality limited by the detector, rather than by the optics. The different colors correspond to the different fields listed in Table 1.

The scene is imaged on a 2D detector. As we use the pushbroom configuration, our detector is an area sensor featuring spatial information in one dimension and spectral information in the other dimension. Owing to the use of freeform optics, the nominal optical design over-achieves the mission requirements. Indeed, while we were targeting a spatial resolution of 20 nm and a spatial resolution of 5 km at nadir, our nominal optical design features a spectral resolution of 13 nm and a spatial resolution of 2.6 km at nadir. The image quality is very good, as for all fields, RMS spot radii almost equal the Airy disk sizes. This means that we achieve diffraction-limited performance over the extremely wide field of view of 120°, as illustrated at a wavelength of 1700 nm by the spot diagram (Table 1, Figure 10) and the modulation transfer function (Figure 11). In addition, we ensured that this diffraction-limited performance is also achieved for all the other wavelengths between 1100 and 1700 nm. Therefore, this WFOV space-based spectrometer surpasses the state-of-the-art in terms of field of view and spatial resolution, and fills a gap in the scientific literature in terms of spectral range.

## 4. Discussion

### 4.1. Technical Comparison of Our Instrument with the State-of-the-Art

We targeted the development of a novel WFOV space-based spectrometer for the monitoring of the three most prominent greenhouse gases in the atmosphere: water vapor, carbon dioxide and methane. These three gases feature spectral lines between 1100 and 1700 nm, and this spectral region is not yet covered by the current state-of-the-art. We intend to fill this gap with our instrument, providing complementary information for a better understanding of the greenhouse gases impacting climate change.

Pushbroom, non-scanning, wide-field-of-view instruments, such as OMI [27], its follow-on TROPOMI [29], the future UVN [33] and UVNS instruments [34], have inspired the design of our own spectrometer. Like the TROPOMI telescope, we use two freeform mirrors featuring an extremely wide field of view, enabling daily global coverage. However, our telescope features a FOV of 120°, which is the widest field of view that has ever been achieved for a space-based telescope (unless TROPOMI whose FOV is approximately 108°), and that almost reaches Earth observation from limb to limb from an altitude of about 700 km. Using such a wide field of view has some costs: (1) it is more challenging to achieve a high spatial resolution, in comparison to narrow-field-of-view instruments, (2) barrel distortion on the image increases with the field angle. However, owing to the use of freeform optics technology, which enables improving image quality and achieving high spatial resolution on ground, our spatial resolution equals 2.6 km × 2.6 km, which is about 2.7 times better than TROPOMI. Therefore, our design not only fills a gap from the spectral point of view, but also surpasses previous instruments in terms of field of view and spatial resolution.

Another point of comparison is the compactness of the design. Recently, the need for compact spectrometers has been put forward for enhanced climate change and/or pollution monitoring, as highlighted, e.g., by the CHAPS instrument [31]. This instrument serves a different purpose than the previously discussed spectrometer, as its goal is to monitor local pollution in urban areas. Hence, the footprint is at the scale of a city, and the field of view is quite narrow (15°). However, the idea of a compact design is important, as it enables the use of low-cost small satellites, such as CubeSats. For example, the CHAPS instrument is a 4U payload, and can be integrated into a 6U CubeSat platform. In comparison to CHAPS, our spectrometer is more compact, fitting inside only 1U. In the longer term, the use of several CubeSats would allow using a constellation of satellites for the same cost as a large-scale space mission, and with the benefit of a much higher scientific yield.

### 4.2. Towards a Proof-of-Concept Demonstrator Setup

The first next steps focus on the realization of a demonstrator setup for the telescope system. This includes an extensive tolerance analysis, manufacturing of the freeform mirrors and the validation of its performance in a laboratory test setup. A first initial tolerance analysis indicated a probability exceeding 90% of achieving the nominal performance for the telescope. The two mirrors can be made of oxygen-free high thermal conductivity (OFHC) copper, and the freeform shape composed of XY polynomials can be manufactured using ultraprecision diamond tooling.

As our telescope achieves RMS spot sizes in the order of 30 µm or less at the spectrometer slit, it would be compatible with other spectrometers operating with wavelengths longer than 1700 nm, while keeping a diffraction-limited design for the telescope. In particular, the thermal infrared region (8–14 µm) could be of interest for the monitoring of trace gases in the atmosphere and highlighting the direct link between greenhouse gases and the outgoing longwave radiation. While the telescope could remain the same—which is the reason why we are going to manufacture a demonstrator setup for this part first—the spectrometer part would most probably need to be re-designed if there is a need to operate at longer wavelengths.

Future research is also needed on the custom grating and custom detector. To maintain the excellent optical performance, some design modifications might be required in view of complying with the manufacturability constraints for this grating and detector. However, the requirements for the grating and detector are feasible. Indeed, the grating is only 120 lines/mm, giving a spectral resolution of 13 nm. To achieve the spatial resolution of 2.6 km, the acquisition time should be 370 ms or less, considering the satellite is moving forward at the speed of about 7 km/s. In addition, the detector would need 933 pixels of 30 µm in the spatial direction and 83 pixels in the spectral direction. The whole detector size would be approximately 28 mm in the spatial direction and 2.5 mm in the spectral direction.

### 4.3. Future Perspectives

As a short-term perspective, future work will include: (1) a detailed radiometric budget using radiative transfer simulations, (2) a radiometric calibration using internal light sources, similarly to the state-of-the-art [45], (3) optimization of hyperspectral processing algorithms (machine learning) to retrieve information on greenhouse gas concentrations on the measured spectra and (4) an end-to-end simulation including the in-orbit operation mode.

As a long-term perspective, we foresee launching the spectrometer on board a small satellite at a Low-Earth-Orbit altitude of 700 km. The satellite could either be a CubeSat companion to the Earth Climate Observatory (ECO), or a SmallSat platform (such as a PROBA satellite) that will carry the spectrometer to sense greenhouse gases, together with the same payload of the ECO satellite, including a WFOV radiometer to measure total incoming solar radiation and total outgoing terrestrial radiation, a WFOV shortwave camera to measure the reflected solar radiation and a WFOV longwave camera to measure the outgoing longwave radiation. These four instruments can operate in synergy to provide measurements of the two most fundamental essential climate variables from space: the greenhouse gases and the Earth’s radiation budget, with the ultimate objective of delivering greatly enhanced climate change monitoring. In addition, the use of CubeSat or SmallSat satellite platforms holds promise for a faster development time at a reduced cost. This means that for the same cost for a large space mission, we could have multiple copies of this satellite, i.e., a constellation of satellites, leading to improved monitoring of our planet.

## 5. Summary and Conclusions

In this paper, we have discussed the in-house design of a space-based instrument composed of a telescope and a spectrometer, aiming to observe the Earth with an extremely wide field of view of 120°, in view of monitoring the three most prominent greenhouse gases in the Earth’s atmosphere: water vapor, carbon dioxide and methane. These three gases feature spectral lines between 1100 and 1700 nm, which are not covered by the state-of-the-art wide-field-of-view spaceborne spectrometers. We intend to fill spectral gap, by collecting and measuring the reflection spectra in this wavelength region, containing the spectral fingerprints of these greenhouse gases.

Our full instrument includes a total of five freeform mirrors and a reflective grating. Freeform optics allows achieving high image quality, whilst minimizing the number of mirrors and maximizing the field of view. Our instrument features a FOV of 120°, which is the widest field of view that has ever been realized for a space-based telescope, and that can almost achieve Earth observation from limb to limb from an altitude of about 700 km. Our spatial resolution of 2.6 km × 2.6 km is better than the state-of-the-art as well. Our design does not only fill a gap in the spectral range, but also delivers better results in terms of field of view and spatial resolution. We therefore feel confident that it is a candidate for future space missions that target enhanced monitoring of climate change.

As future work, a demonstrator setup will be manufactured and characterized in the laboratory. Additionally, algorithms will be developed to assess the radiometric budget, along with in-orbit calibration, the retrieval of greenhouse gas concentrations on the measured spectra and an end-to-end simulation. These steps will pave the path to the future ECO space mission, aiming to improve climate change monitoring from space.

## Figures and Tables

**Table 1:** RMS spot radii at 1700 nm over the full field of view. For each field, the root-mean-square size of the spot is smaller than the Airy disk (radius = 14.98 µm), implying a diffraction-limited design. The results are shown for an equiangular sampling over the half field of view. Results are symmetrical with the other half of the field of view.

	Half FOV (°)	RMS Spot Radius (μm)
1st field	0.00	8.755
2nd field	5.46	7.276
3rd field	10.91	4.961
4th field	16.36	4.773
5th field	21.82	5.109
6th field	27.27	4.708
7th field	32.73	4.428
8th field	38.18	5.085
9th field	43.63	5.900
10th field	49.09	6.000
11th field	54.55	6.988
12th field	60.00	14.924
